# Supplementary material for: Temporal comparison and predictors of fish species abundance and richness on undisturbed coral reef patches
Source: PeerJ. 2015 Dec 1;3:e1459. doi: 10.7717/peerj.1459 (PMC4671157; doi:10.7717/peerj.1459)
Supplement: Supplemental Information 1 — Tables S1, S2, S3, S4 Figures S1, S2, S3, S4, S5, S6. [file peerj-03-1459-s001.docx]

**Temporal comparison and predictors of fish species abundance and richness on undisturbed coral reef patches**

Elena L. E. Wagner^†^, Dominique G. Roche^†^, Sandra A. Binning^†^*, Sharon Wismer & Redouan Bshary

*Université de Neuchâtel, Institut de Biologie, Émile-Argand 11, 2000 Neuchâtel, Switzerland*

^†^ Joint first authors

*Corresponding author: binningsandra@gmail.com

**Electronic Supplementary Material**

**Main effects and interaction terms included in the full models:**

Fish abundance:

Year

Percent live coral cover (PLCC)

Patch volume (PV)

I(PV^2^)

Depth
Percent caves (PC)

Percent holes (PH)

*Labroides dimidiatus* abundance (Ldim)

PLCC:Depth

PLCC:PC

PV:PC

D:PC

D:Ldim

PC:PH

PH:Ldim

Fish species richness:

Year

Percent live coral cover (PLCC)

Patch volume (PV)

I(PV^2^)

Depth
Percent caves (PC)

Percent holes (PH)

*Labroides dimidiatus* abundance (Ldim)

PLCC:D

PLCC:PC

PV:PC

PV:PH

PC:Ldim

PH:Ldim**Supplementary tables**

**Table S1.** GPS coordinates of the 47 patch reefs examined in this study. Patch = patch ID, Date = date GPS coordinates were recorded. This table is also available in .csv format on the data repository figshare: <http://dx.doi.org/10.6084/m9.figshare.1335775>

| **Patch** | **Date** | **GPS Coordinates** | **Latitude** | **Longitude** |
| --- | --- | --- | --- | --- |
| 1 | 21/10/2007 | N27 47.538 E34 12.943 | 27.7923 | 34.21571667 |
| 2 | 21/10/2007 | N27 47.528 E34 12.947 | 27.79213333 | 34.21578333 |
| 3 | 21/10/2007 | N27 47.526 E34 12.961 | 27.7921 | 34.21601667 |
| 4 | 21/10/2007 | N27 47.521 E34 12.956 | 27.79201667 | 34.21593333 |
| 5 | 21/10/2007 | N27 47.513 E34 12.964 | 27.79188333 | 34.21606667 |
| 6 | 21/10/2007 | N27 47.504 E34 12.976 | 27.79173333 | 34.21626667 |
| 7 | 21/10/2007 | N27 47.496 E34 12.984 | 27.7916 | 34.2164 |
| 8 | 21/10/2007 | N27 47.491 E34 12.981 | 27.79151667 | 34.21635 |
| 9 | 21/10/2007 | N27 47.489 E34 12.978 | 27.79148333 | 34.2163 |
| 10 | 21/10/2007 | N27 47.488 E34 12.987 | 27.79146667 | 34.21645 |
| 11 | 21/10/2007 | N27 47.481 E34 12.989 | 27.79135 | 34.21648333 |
| 13 | 21/10/2007 | N27 47.457 E34 12.984 | 27.79095 | 34.2164 |
| 14 | 21/10/2007 | N27 47.461 E34 12.990 | 27.79101667 | 34.2165 |
| 15 | 21/10/2007 | N27 47.463 E34 12.995 | 27.79105 | 34.21658333 |
| 16 | 21/10/2007 | N27 47.461 E34 12.996 | 27.79101667 | 34.2166 |
| 17 | 21/10/2007 | N27 47.458 E34 12.999 | 27.79096667 | 34.21665 |
| 19 | 21/10/2007 | N27 47.447 E34 13.004 | 27.79078333 | 34.21673333 |
| 20 | 21/10/2007 | N27 47.442 E34 13.000 | 27.7907 | 34.21666667 |
| 21 | 21/10/2007 | N27 47.431 E34 12.998 | 27.79051667 | 34.21663333 |
| 22 | 21/10/2007 | N27 47.429 E34 12.999 | 27.79048333 | 34.21665 |
| 23 | 21/10/2007 | N27 47.424 E34 13.003 | 27.7904 | 34.21671667 |
| 24 | 21/10/2007 | N27 47.417 E34 13.004 | 27.79028333 | 34.21673333 |
| 25 | 21/10/2007 | N27 47.419 E34 13.017 | 27.79031667 | 34.21695 |
| 26 | 21/10/2007 | N27 47.417 E34 13.028 | 27.79028333 | 34.21713333 |
| 27 | 22/10/2007 | N27 47.405 E34 13.033 | 27.79008333 | 34.21721667 |
| 28 | 22/10/2007 | N27 47.395 E34 13.030 | 27.78991667 | 34.21716667 |
| 29 | 22/10/2007 | N27 47.382 E34 13.040 | 27.7897 | 34.21733333 |
| 30 | 22/10/2007 | N27 47.383 E34 13.063 | 27.78971667 | 34.21771667 |
| 31 | 22/10/2007 | N27 47.372 E34 13.066 | 27.78953333 | 34.21776667 |
| 32 | 22/10/2007 | N27 47.360 E34 13.065 | 27.78933333 | 34.21775 |
| 33 | 22/10/2007 | N27 47.358 E34 13.081 | 27.7893 | 34.21801667 |
| 34 | 22/10/2007 | N27 47.365 E34 13.063 | 27.78941667 | 34.21771667 |
| 35 | 22/10/2007 | N27 47.351 E34 13.082 | 27.78918333 | 34.21803333 |
| 36 | 22/10/2007 | N27 47.375 E34 13.047 | 27.78958333 | 34.21745 |
| 37 | 21/10/2007 | N27 47.452 E34 13.010 | 27.79086667 | 34.21683333 |
| 38 | 21/10/2007 | N27 47.444 E34 13.017 | 27.79073333 | 34.21695 |
| 39 | 21/10/2007 | N27 47.515 E34 12.974 | 27.79191667 | 34.21623333 |
| 251 | 21/10/2007 | N27 47.424 E34 13.019 | 27.7904 | 34.21698333 |
| 261 | 21/10/2007 | N27 47.413 E34 13.028 | 27.79021667 | 34.21713333 |
| 271 | 22/10/2007 | N27 47.406 E34 13.028 | 27.7901 | 34.21713333 |
| 281 | 22/10/2007 | N27 47.388 E34 13.031 | 27.7898 | 34.21718333 |
| 311 | 22/10/2007 | N27 47.370 E34 13.076 | 27.7895 | 34.21793333 |
| 321 | 22/10/2007 | N27 47.360 E34 13.067 | 27.78933333 | 34.21778333 |
| 341 | 22/10/2007 | N27 47.368 E34 13.096 | 27.78946667 | 34.21826667 |
| 352 | 22/10/2007 | N27 47.345 E34 13.078 | 27.78908333 | 34.21796667 |
| 371 | 21/10/2007 | N27 47.464 E34 13.002 | 27.79106667 | 34.2167 |

**Table S2.** Mean abundance (± SD; one standard deviation) of coral reef fish species recorded at the 47 patch reefs in 1997 and 2007. Category groups fish species as resident (R), facultative visitor (FV), visitor (V) or undefined (U); see main text for definitions. This table is also available in .csv format on the data repository figshare: <http://dx.doi.org/10.6084/m9.figshare.1335775>

| **Family** | **Common family name** | **Species name** | **Abundance 1997**  **(mean ± SD)** | **Abundance 2007**  **(mean ± SD)** | **Category** |
| --- | --- | --- | --- | --- | --- |
| Acanthuridae | Surgeonfish | *Acanthurus nigrofuscus* | 65 ± 1.00 | 99.2 ± 15.07 | R |
|  |  | *Ctenochaetus striatus* | 16.67 ± 1.53 | 49.4 ± 5.94 | R |
|  |  | *Naso literatus* | 0.33 ± 0.58 | 0.6 ± 0.89 | V |
|  |  | *Zebrasoma veliferum* | 12.33 ± 4.93 | 14.6 ± 6.58 | R |
|  |  | *Zebrasoma xanthurum* | / | 2.6 ± 0.55 | R |
| Apogonidae | Cardinalfish | *Apogon cyanosoma* | 127 ± 21.17 | 442.4 ± 120.43 | R |
|  |  | *Apogon exostigma* | / | 149.4 ± 88.04 | R |
|  |  | *Apogon fraenatus* | 148.33 ± 69.21 | 0.4 ± 0.89 | R |
|  |  | *Apogon kallopterus* | / | 31.6 ± 5.13 | R |
|  |  | *Apogon nigrofasciatus* | / | 25 ± 9.35 | R |
|  |  | *Cheilodipterus lineatus* | / | 30.4 ± 6.69 | R |
|  |  | *Cheilodipterus quinquelineatus* | 77.67 ± 18.56 | 44.6 ± 9.45 | R |
|  |  | "Unidentified Cardinalfish a"^a^ | 0.33 ± 0.58 | 0.2 ± 0.45 | R |
|  |  | "Unidentified Cardinalfish b" ^a^ | 1 ± 1.00 | / | R |
|  |  | "Unidentified Cardinalfish c" ^a^ | 16.33 ± 6.66 | / | R |
|  |  | "Unidentified Cardinalfish d" ^a^ | 0.67 ± 0.58 | / | R |
| Balistidae | Triggerfish | *Balistapus undulatus* | 4 ± 1.00 | 4.2 ± 1.79 | FV |
|  |  | *Odonus niger* | 2.67 ± 2.52 | 1.2 ± 1.30 | V |
|  |  | *Pseudobalistes fuscus* | / | 0.2 ± 0.45 | V |
|  |  | *Rhinecanthus assasi* | 1.67 ± 1.53 | 4.8 ± 3.11 | V |
|  |  | *Sufflamen albicaudatus* | 12.33 ± 2.52 | 16.4 ± 2.51 | R |
| Blennidae | Blenny | *Aspidontus taeniatus* | 0.33 ± 0.58 | / | R |
|  |  | *Plagiotremus rhinorhynchos* | 1.67 ± 1.53 | 0.2 ± 0.45 | R |
|  |  | *Plagiotremus tapeinosoma* | 16.67 ± 5.69 | 7.6 ± 3.78 | R |
| Caesionidae | Fusilier | *Caesio lunaris* | 0.33 ± 0.58 | 4.6 ± 8.65 | V |
|  |  | *Caesio suenicus* | 2 ± 3.46 | 3.8 ± 8.50 | V |
|  |  | *Pterocaesio chrysozoma* | / | 10.4 ± 12.44 | V |
|  |  | Mix of fusiliers^b^ | / | 5.8 ± 10.23 | V |
| Chaetodontidae | Butterflyfish | *Chaetodon auriga* | 10 ± 4.00 | 6.6 ± 2.51 | FV |
|  |  | *Chaetodon austriacus* | 17.33 ± 0.58 | 16.4 ± 8.91 | FV |
|  |  | *Chaetodon fasciatus* | 9.67 ± 1.53 | 10.8 ± 3.27 | FV |
|  |  | *Chaetodon melannotus* | 2.67 ± 3.06 | 2 ± 1.22 | FV |
|  |  | *Chaetodon paucifasciatus* | 24 ± 4.00 | 34.4 ± 7.40 | FV |
|  |  | *Chaetodon semilarvatus* | / | 2 ± 0.00 | R |
|  |  | *Heniochus intermedius* | 20.67 ± 1.15 | 15.2 ± 0.84 | R |
|  |  | *Megaprotodon trifasialis* | / | 0.6 ± 0.55 | FV |
| Cirrhitidae | Hawkfish | *Cirrhitichtys oxycephalus* | / | 1.8 ± 0.45 | U |
|  |  | *Paracirrhites forsteri* | 5.33 ± 1.53 | 14 ± 2.12 | FV |
| Diodontidae | Porcupinefish | *Diodon hystrix* | 0.33 ± 0.58 | 0.4 ± 0.55 | V |
| Gobiidae | Goby | *Ptereleotris microlepis* | / | 1.6 ± 2.61 | R |
| Grammistidae | Soapfish | *Grammistes sexlineatus* | / | 0.8 ± 0.45 | R |
| Holocentridae | Squirrelfish and soldierfish | *Adioryx diadema* | 29.67 ± 2.52 | 163 ± 28.27 | R |
|  |  | *Flammeo sammara* | 0.67 ± 0.58 | 1.2 ± 0.84 | R |
|  |  | *Myripristis murdjan* | 39.33 ± 2.52 | 56.4 ± 6.80 | R |
|  |  | *Sargocentron caudimaculatum* | 15.67 ± 0.58 | 22 ± 1.87 | R |
| Labridae | Wrasse | *Anampses meleagrides* | / | 1.4 ± 0.55 | FV |
|  |  | *Anampses twistii* | / | 0.4 ± 0.55 | V |
|  |  | *Bodianus anthioides* | 2 ± 2.65 | 5 ± 1.58 | FV |
|  |  | *Cheilinus abudjubbe* | / | 1.2 ± 1.30 | V |
|  |  | *Cheilinus diagrammus* | / | 1.4 ± 1.95 | V |
|  |  | *Cheilinus lunatus* | / | 0.8 ± 0.84 | V |
|  |  | *Cheilinus mentalis* | / | 0.2 ± 0.45 | V |
|  |  | *Cheilio normis* | / | 0.2 ± 0.45 | V |
|  |  | *Coris aygula* | 0.67 ± 0.58 | 3.4 ± 0.89 | V |
|  |  | *Coris caudimacula* | / | 0.6 ± 0.55 | V |
|  |  | *Coris variegata* | / | 1.8 ± 0.84 | V |
|  |  | *Gomphosus caeruleus* | 23 ± 6.08 | 27.4 ± 7.09 | V |
|  |  | *Halichoeres hortulanus* | / | 0.6 ± 0.89 | FV |
|  |  | *Halichoeres scaplularis* | 117 ± 6.56 | 0.8 ± 1.10 | R |
|  |  | *Labroides dimidiatus* | 21.33 ± 3.21 | 15 ± 3.46 | R |
|  |  | *Pseudocheilinus hexataenia* | 157.67 ± 27.93 | 78.2 ± 10.71 | R |
|  |  | *Stethojulis albovittata* | / | 2 ± 1.73 | U |
|  |  | *Thalassoma lunare* | 17.33 ± 4.93 | 5.2 ± 0.84 | FV |
|  |  | *Thalassoma rueppellii* | 39.67 ± 10.69 | 123.6 ± 17.21 | V |
|  |  | "Undetermined Labridae"^a^ | 0.33 ± 0.58 | / | U |
|  |  | "Labridae"^b^ | 2.67 ± 0.58 | 0.4 ± 0.55 | U |
| Lethrinidae | Emperor | *Lethrinus xanthochilus* | / | 0.2 ± 0.45 | V |
|  |  | *Monotaxis grandoculis* | 1.67 ± 1.15 | 10.2 ± 15.16 | FV |
|  |  | “Undetermined Emperor”^a^ | / | 3.4 ± 2.30 | V |
| Monacanthidae | Filefish | *Cantherhines pardalis* | / | 4.8 ± 2.17 | V |
| Mullidae | Goatfish | *Parupeneus forsskali* | 17.33 ± 11.93 | 21 ± 13.19 | V |
|  |  | *Parupeneus macronema* | 1 ± 1.73 | 10 ± 4.85 | V |
|  |  | *Mulloides flavolineatus* | / | 6.4 ± 4.88 | V |
| Nemipteridae | Spinecheek | *Scolopsis ghanam* | 4 ± 6.08 | 5.4 ± 1.67 | V |
| Ostraciidae | Trunkfish | *Ostracion cyanurus* | 2.33 ± 1.15 | 3.4 ± 2.30 | V |
| Pempheridae | Sweeper | *Pempheris schwenkii* | / | 2 ± 3.39 | R |
| Pomacanthidae | Angelfish | *Centropyge multispinis* | 26.33 ± 3.21 | 34.6 ± 3.78 | R |
|  |  | *Pygoplites diacanthus* | 1 ± 1.00 | 2.6 ± 1.82 | FV |
| Pomacentridae | Damselfish | *Abudefduf saxatilis* | / | 14 ± 10.93 | V |
|  |  | *Amblyglyphidodon flavilatus* | / | 0.2 ± 0.45 | FV |
|  |  | *Amblyglyphidodon leucogaster* | 75 ± 19.08 | 214 ± 29.94 | R |
|  |  | *Amphiprion bicinctus* | 8.33 ± 1.53 | 13.4 ± 2.07 | R |
|  |  | *Chromis dimidiata* | 429.33 ± 51.05 | 670.4 ± 105.03 | R |
|  |  | *Chromis ternatensis* | 574.67 ± 53.26 | 223.8 ± 53.71 | R |
|  |  | *Chromis viridis* | 2024.33 ± 125.17 | 684.6 ± 168.97 | R |
|  |  | *Chromis weberi* | 86.33 ± 14.57 | 78.8 ± 46.86 | R |
|  |  | *Chrysiptera unimaculata* | 0.67 ± 0.58 | / | R |
|  |  | *Dascyllus aruanus* | 35.33 ± 9.50 | 15 ± 1.41 | R |
|  |  | *Dascyllus marginatus* | 79 ± 11.79 | 103.2 ± 7.46 | R |
|  |  | *Dascyllus trimaculatus* | 52.67 ± 15.31 | 80.4 ± 7.89 | R |
|  |  | *Neopomacentrus miryae* | 37.67 ± 5.51 | 141.2 ± 28.37 | R |
|  |  | *Paraglyphidodon melas* | 1.67 ± 1.15 | / | R |
|  |  | *Plectroglyphidon lacrymatus* | 9 ± 2.65 | 13.8 ± 2.59 | R |
|  |  | *Pomacentrus sulfureus* | 2 ± 0.00 | 2 ± 0.71 | R |
|  |  | *Pomacentrus trichourus* | 228.67 ± 10.41 | 252.6 ± 17.11 | R |
|  |  | *Pomacentrus trilineatus* | 3.33 ± 0.58 | 0.4 ± 0.55 | R |
|  |  | *Stegaster nigricans* | / | 0.2 ± 0.45 | R |
| Priacanthidae | Bigeye | *Priacanthus hamrur* | 1 ± 1.73 | / | R |
| Pseudochromidae | Dottyback | *Pseudochromis fridmani* | 196.33 ± 42.25 | 132.8 ± 13.97 | R |
|  |  | *Pseudochromis olivaceus* | 46.67 ± 8.50 | 50 ± 4.95 | R |
|  |  | *Pseudochromis springeri* | 18 ± 1.73 | 32.4 ± 4.56 | R |
| Scaridae | Parrotfish | *Calotomus viridescens* | / | 6.6 ± 2.07 | V |
|  |  | *Clorurus gibbus* | / | 0.2 ± 0.45 | V |
|  |  | *Hipposcarus harid* | 2.33 ± 2.08 | 10.2 ± 3.96 | V |
|  |  | *Scarus ferrugineus* | / | 3.6 ± 1.95 | V |
|  |  | *Scarus frenatus* | / | 0.8 ± 1.30 | V |
|  |  | *Scarus niger* | / | 1.2 ± 1.10 | V |
|  |  | *Scarus sordidus* | 2 ± 2.00 | 42.8 ± 7.79 | V |
|  |  | *“*Scaridae*”*^b^ | 19.33 ± 7.23 | 1 ± 1.22 | V |
| Scorpaenidae | Scorpionfish | *Pterois radiata* | / | 4 ± 2.00 | FV |
|  |  | *Pterois volitans* | 12 ± 2.00 | 8.4 ± 0.89 | FV |
|  |  | *Sebastes cyanostigma* | / | 1.6 ± 1.52 | FV |
| Serranidae | Groupers and Anthiases | *Aetaloperca rogaa* | 1 ± 0.00 | / | R |
|  |  | *Anthias squamipinnis* | 1524 ± 53.67 | 974.4 ± 115.34 | R |
|  |  | *Anthias taenatus* | 0.67 ± 1.15 | 1.2 ± 1.64 | U |
|  |  | *Cephalopholis argus* | / | 0.4 ± 0.55 | FV |
|  |  | *Cephalopholis hemistiktos* | 40.33 ± 2.31 | 38.2 ± 3.96 | R |
|  |  | *Cephalopholis miniata* | 13.67 ± 2.31 | 10.4 ± 2.61 | R |
|  |  | *Epinephelus fasciatus* | 8.33 ± 3.21 | 6.2 ± 1.79 | FV |
| Siganidae | Rabbitfish | *Siganus luridus* | / | 16.8 ± 4.76 | FV |
|  |  | *Siganus rivulatus* | / | 2.8 ± 1.30 | V |
|  |  | *Ostracion cubicus* | / | 0.2 ± 0.45 | V |
| Tetraodontidae | Pufferfish | *Canthigaster coronata* | 0.33 ± 0.58 | 0.4 ± 0.55 | U |
|  |  | *Canthigaster pygmaea* | / | 0.2 ± 0.45 | U |
|  |  | *Arothron diadematus* | 1.33 ± 1.53 | 1.2 ± 1.64 | V |
| Unidentified juveniles |  | "Juveniles"^b^ | 250 ± 45.83 | 25.4 ± 43.14 | U |
| Unidentified adults |  | "Unidentified a"^a^ | 0.33 ± 0.58 | / | R |
|  |  | "Unidentified b" ^a^ | 4.67 ± 6.35 | / | U |
|  |  | "Unidentified c" ^a^ | 0.33 ± 0.58 | / | U |
|  |  | "Unidentified Species"^b^ | 1 ± 1.00 | 0.8 ± 0.45 | U |

^a^ Indicates that a species could not be identified, but differentiated visually from other species.

^b^ Includes more than one species that could neither be identified nor clearly differentiated from other species.

**Table S3.** (This is an extended version of Table 2a) Predictors and interaction terms included in the best models explaining variation in fish abundance among patch reefs (n=47) and years (1997 vs. 2007). The model averaged parameters estimates (β), unconditional standard errors (SE), 95% confidence interval (95% CI), and the normalized Akaike weight (*w*_ip_) for each predictor are shown. Also indicated are the models in which predictors were included. Predictors are ordered by *w*_ip_; those for which the 95% CI does not overlap zero are indicated in bold. All models include a constant and patch ID was specified as a random factor.

| **Predictor** | **Model Rank** | | | | | | | | | | | | | | | |
| --- | --- | --- | --- | --- | --- | --- | --- | --- | --- | --- | --- | --- | --- | --- | --- | --- |
|  | **1** | **2** | **3** | **4** | **5** | **6** | **7** | **8** | **9** | **10** | **11** | **12** | **13** | **14** | **15** | **16** |
| Intercept | x | x | x | x | x | x | x | x | x | x | x | x | x | x | x | x |
| **PCa** | x | x | x | x | x | x | x | x | x | x | x | x | x | x | x | x |
| **PLCC** | x | x | x | x | x | x | x | x | x | x | x | x | x | x | x | x |
| **PV** | x | x | x | x | x | x | x | x | x | x | x | x | x | x | x | x |
| **PV2** | x | x | x | x | x | x | x | x | x | x | x | x | x | x | x | x |
| **PCa:PLCC** | x | x | x | x | x | x | x | x | x | x | x | x | x | x | x | x |
| **PCa:PV** | x | x | x | x | x | x | x | x | x | x | x | x | x | x | x | x |
| Ldim | x | x |  |  | x |  | x | x |  | x | x | x | x | x | x |  |
| Year | x |  | x |  | x | x |  | x | x |  |  | x |  |  | x |  |
| PH |  |  |  |  | x |  | x |  | x |  | x | x | x | x | x |  |
| Depth |  |  |  |  |  | x |  | x |  | x |  |  |  |  |  | x |
| PH:Ldim |  |  |  |  |  |  |  |  |  |  | x | x | x |  |  |  |
| PCa:PH |  |  |  |  |  |  |  |  |  |  |  |  | x | x | x |  |
| **df** | 11 | 10 | 10 | 9 | 12 | 11 | 11 | 12 | 11 | 11 | 12 | 13 | 13 | 12 | 13 | 10 |
| **logLik** | -75.77 | -77.14 | -77.15 | -79.28 | -75.72 | -77.05 | -77.08 | -75.77 | -77.09 | -77.14 | -75.87 | -74.81 | -75.22 | -76.57 | -75.24 | -79.20 |
| **AICc** | 176.8 | 176.9 | 176.9 | 178.7 | 179.3 | 179.3 | 179.4 | 179.4 | 179.4 | 179.5 | 179.6 | 180.2 | 181 | 181 | 181 | 181 |
| **Delta AICc** | 0 | 0.16 | 0.17 | 1.93 | 2.53 | 2.56 | 2.61 | 2.63 | 2.64 | 2.73 | 2.82 | 3.4 | 4.21 | 4.22 | 4.27 | 4.28 |
| ***w*_im_** | 0.168 | 0.155 | 0.154 | 0.064 | 0.047 | 0.047 | 0.045 | 0.045 | 0.045 | 0.043 | 0.041 | 0.031 | 0.02 | 0.02 | 0.02 | 0.02 |

| **Predictor** | **Model Rank** | | | | | |
| --- | --- | --- | --- | --- | --- | --- |
|  | **17** | **18** | **B** | **SE** | **95% CI** | ***w*_ip_** |
| Intercept | x | x | 4.85 | 0.15 | 4.56 to 5.15 | 1.00 |
| **PCa** | x | x | **0.35** | **0.12** | **0.12 to 0.58** | **1.00** |
| **PLCC** | x | x | **0.23** | **0.06** | **0.11 to 0.36** | **1.00** |
| **PV** | x | x | **1.05** | **0.14** | **0.78 to 1.32** | **1.00** |
| **PV2** | x | x | **-0.42** | **0.11** | **-0.63 to -0.21** | **1.00** |
| **PCa:PLCC** | x | x | **0.21** | **0.06** | **0.08 to 0.33** | **1.00** |
| **PCa:PV** | x | x | **0.41** | **0.13** | **0.16 to 0.66** | **1.00** |
| Ldim |  |  | 0.10 | 0.06 | -0.01 to 0.22 | 0.63 |
| Year |  | x | -0.16 | 0.09 | -0.33 to 0.01 | 0.57 |
| PH | x | x | -0.03 | 0.08 | -0.18 to 0.13 | 0.31 |
| Depth |  |  | -0.02 | 0.08 | -0.18 to 0.15 | 0.15 |
| PH:Ldim |  |  | 0.08 | 0.05 | -0.02 to 0.17 | 0.09 |
| PCa:PH |  | x | 0.09 | 0.09 | -0.09 to 0.28 | 0.08 |
| **df** | 10 | 12 |  |  |  |  |
| **logLik** | -79.21 | -76.75 |  |  |  |  |
| **AICc** | 181.1 | 181.4 |  |  |  |  |
| **delta AICc** | 4.31 | 4.59 |  |  |  |  |
| ***w*_im_** | 0.019 | 0.017 |  |  |  |  |

**Abbreviations :**

df: degrees of freedom

Depth: depth below the surface

Ldim: *L. dimidiatus* abundance

logLik: log likelihood

PCa : percent caves

PLCC: percent live coral cover

PH: percent holes

PV: patch volume

PV2: (patch volume)^2^

Year: survey year

| **Predictor** | **Model Rank** | | | | | | | | | | | | | | | |
| --- | --- | --- | --- | --- | --- | --- | --- | --- | --- | --- | --- | --- | --- | --- | --- | --- |
|  | **19** | **20** | **21** | **22** | **23** | **24** | **25** | **26** | **27** | **28** | **29** | **30** | **B** | **SE** | **95% CI** | ***w*_ip_** |
| Intercept | x | x | x | x | x | x | x | x | x | x | x | x | 20.44 | 0.98 | 18.51 to 22.37 | 1.00 |
| **PLCC** | x | x | x | x | x | x | x | x | x | x | x | x | **1.23** | **0.41** | **0.44 to 2.03** | **1.00** |
| **Ldim** | x | x | x | x | x | x | x | x | x | x | x | x | **1.75** | **0.39** | **0.99 to 2.52** | **1.00** |
| **Year** | x | x | x | x | x | x | x | x | x | x | x | x | **8.34** | **0.92** | **6.53 to 10.15** | **1.00** |
| **PV** | x | x | x | x | x | x | x | x | x | x | x | x | **-2.48** | **0.76** | **-3.97 to -1.00** | **1.00** |
| **PV^2^** | x | x | x | x | x | x | x | x | x | x | x | x | **2.87** | **0.59** | **1.71 to 4.03** | **1.00** |
| **PCa** | x | x | x | x | x | x | x | x | x | x | x | x | **1.68** | **0.78** | **0.15 to 3.20** | **0.96** |
| **PCa:PV** | x | x | x | x |  | x | x |  | x | x | x | x | **2.21** | **0.82** | **0.61 to 3.81** | **0.91** |
| **PCa:PLCC** | x |  |  |  | x | x | x | x | x | x |  | x | **0.88** | **0.40** | **0.10 to 1.67** | **0.77** |
| Depth | x | x | x | x | x |  | x | x | x | x | x |  | -0.77 | 0.54 | -1.83 to 0.29 | 0.70 |
| PH | x |  |  | x |  | x |  |  | x | x | x | x | 0.71 | 0.53 | -0.33 to 1.74 | 0.62 |
| **PH:PV** | x |  |  |  |  | x |  |  |  | x | x |  | **1.60** | **0.65** | **0.33 to 2.87** | **0.55** |
| **PLCC:Depth** |  | x |  | x | x |  |  | x |  | x |  |  | **0.97** | **0.45** | **0.09 to 1.85** | **0.48** |
| PCa:Ldim |  | x |  |  |  | x | x | x |  | x |  |  | 0.69 | 0.70 | -0.68 to 2.05 | 0.21 |
| PH:Ldim | x |  |  |  |  | x |  |  |  | x |  |  | 0.20 | 0.33 | -0.47 to 0.84 | 0.11 |
| **df** | 15 | 13 | 11 | 13 | 12 | 15 | 13 | 13 | 13 | 17 | 13 | 12 |  |  |  |  |
| **logLik** | -247.60 | -250.46 | -253.15 | -250.55 | -251.91 | -247.81 | -250.61 | -250.62 | -250.71 | -244.96 | -250.74 | -252.09 |  |  |  |  |
| **AICc** | 531.3 | 531.5 | 531.5 | 531.6 | 531.7 | 531.8 | 531.8 | 531.8 | 532 | 532 | 532 | 532 |  |  |  |  |
| **Delta AICc** | 3.85 | 3.98 | 4.02 | 4.15 | 4.18 | 4.29 | 4.29 | 4.29 | 4.48 | 4.49 | 4.55 | 4.55 |  |  |  |  |
| ***w*_im_** | 0.019 | 0.018 | 0.017 | 0.016 | 0.016 | 0.015 | 0.015 | 0.015 | 0.014 | 0.014 | 0.013 | 0.013 |  |  |  |  |

| **Predictor** | **Model Rank** | | | | | | | | | | | | | | | |  |  |
| --- | --- | --- | --- | --- | --- | --- | --- | --- | --- | --- | --- | --- | --- | --- | --- | --- | --- | --- |
|  | **1** | **2** | **3** | **4** | **5** | **6** | **7** | **8** | **9** | **10** | **11** | **12** | **13** | **14** | **15** | **16** | **17** | **18** |
| Intercept | x | x | x | x | x | x | x | x | x | x | x | x | x | x | x | x | x | x |
| **PLCC** | x | x | x | x | x | x | x | x | x | x | x | x | x | x | x | x | x | x |
| **Ldim** | x | x | x | x | x | x | x | x | x | x | x | x | x | x | x | x | x | x |
| **Year** | x | x | x | x | x | x | x | x | x | x | x | x | x | x | x | x | x | x |
| **PV** | x | x | x | x | x | x | x | x | x | x | x | x | x | x | x | x | x | x |
| **PV^2^** | x | x | x | x | x | x | x | x | x | x | x | x | x | x | x | x | x | x |
| **PCa** | x | x | x | x | x | x | x |  | x | x | x | x | x | x | x | x | x | x |
| **PCa:PV** | x | x | x | x | x | x | x |  | x | x | x | x | x | x | x | x | x |  |
| **PCa:PLCC** | x | x | x | x |  | x | x |  | x | x | x |  | x | x |  | x | x |  |
| Depth |  | x | x | x | x | x |  | x |  |  | x | x | x | x |  | x | x | x |
| PH | x | x |  | x |  |  | x |  | x |  | x | x |  | x | x | x | x |  |
| **PH:PV** | x | x |  | x |  |  | x |  | x |  | x | x |  | x | x |  | x |  |
| **PLCC:Depth** |  | x | x |  | x |  |  | x |  |  | x | x | x |  |  | x | x | x |
| PCa:Ldim |  |  |  |  |  |  | x |  |  |  | x |  | x | x |  |  |  |  |
| PH:Ldim |  |  |  |  |  |  |  |  | x |  |  |  |  |  |  |  | x |  |
| **df** | 13 | 15 | 13 | 14 | 12 | 12 | 14 | 10 | 14 | 11 | 16 | 14 | 14 | 15 | 12 | 14 | 16 | 11 |
| **logLik** | -248.47 | -246.21 | -249.0 | -247.73 | -250.71 | -250.85 | -248.13 | -253.58 | -248.37 | -252.49 | -245.57 | -248.52 | -248.57 | -247.30 | -251.51 | -248.87 | -246.02 | -253.05 |
| **AICc** | 527.5 | 528.6 | 528.6 | 528.8 | 529.3 | 529.6 | 529.6 | 529.8 | 530.1 | 530.2 | 530.2 | 530.4 | 530.4 | 530.8 | 530.9 | 531.1 | 531.1 | 531.3 |
| **Delta AICc** | 0 | 1.08 | 1.09 | 1.28 | 1.78 | 2.07 | 2.09 | 2.33 | 2.58 | 2.72 | 2.72 | 2.86 | 2.96 | 3.27 | 3.39 | 3.57 | 3.62 | 3.82 |
| ***w*_im_** | 0.13 | 0.076 | 0.076 | 0.069 | 0.054 | 0.046 | 0.046 | 0.041 | 0.036 | 0.034 | 0.034 | 0.031 | 0.03 | 0.025 | 0.024 | 0.022 | 0.021 | 0.019 |

**Table S4.** (This is an extended version of Table 2b) Predictors and interaction terms included in the best models explaining variation in fish species richness among patch reefs (n=47) and years (1997 vs. 2007). The model averaged parameters estimates (β), unconditional standard errors (SE), 95% confidence interval (95% CI), and the normalized Akaike weight (*w*_ip_) for each predictor are shown. Also indicated are the models in which predictors were included. Predictors are in order of importance (*w*_ip_); those for which the 95% CI does not overlap zero are indicated in bold. All models include a constant and patch ID was specified as a random factor.

**Supplementary figures**

**Fig. S1** **A)** The relationship between log fish abundance and percent live coral cover at different levels of the continuous predictor ‘percent caves’ (indicated by the orange vertical bar at the top of each graph with values of percent cave increasing from left to right, bottom to top). Graphs display model predictions obtained with the R package effects (Fox et al. 2014). Predictors are scaled (i.e. a one-unit difference represents a difference of one-standard deviation). The grey area is the 95% confidence interval. Vertical black lines indicate the range of data points used in the model. **B)** The same relationship displayed for the raw, untransformed data, for each year sampled (1997 and 2007). N.B. Panels in this figure represent different levels of percent live coral cover rather than percent caves.

**A)**


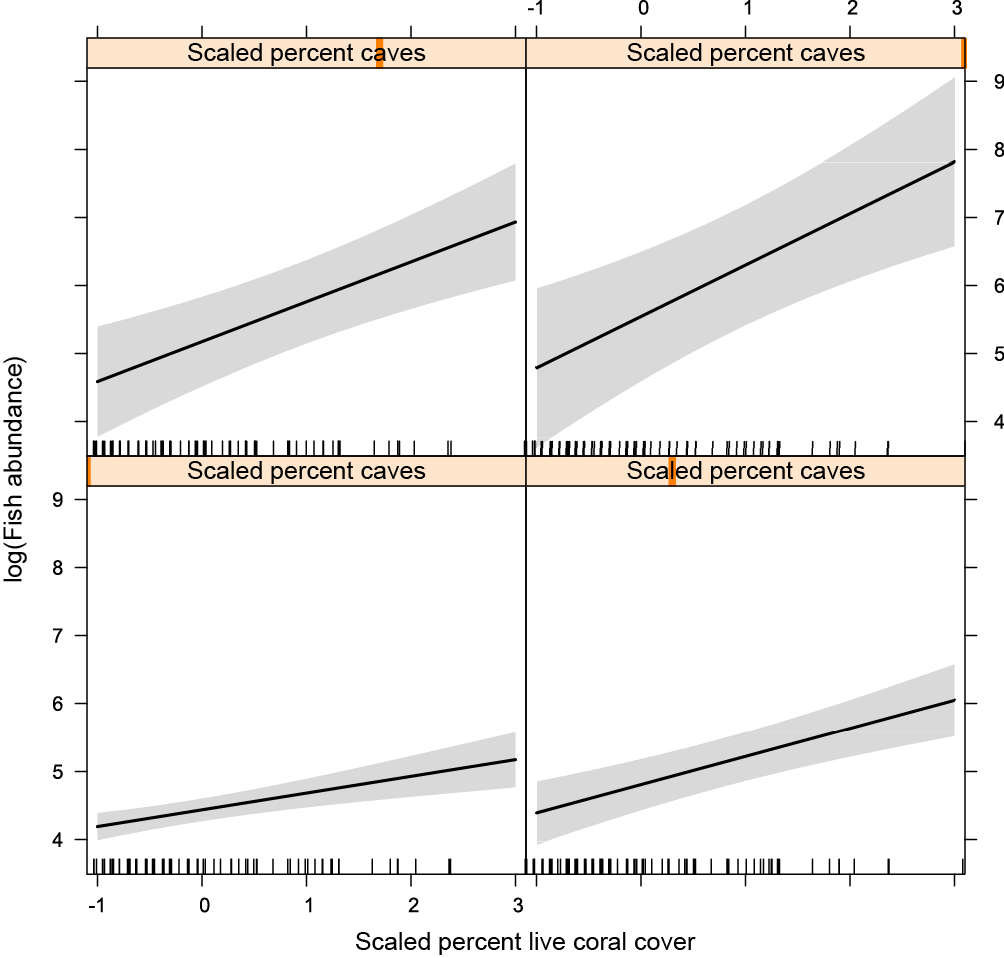


**B)**

**
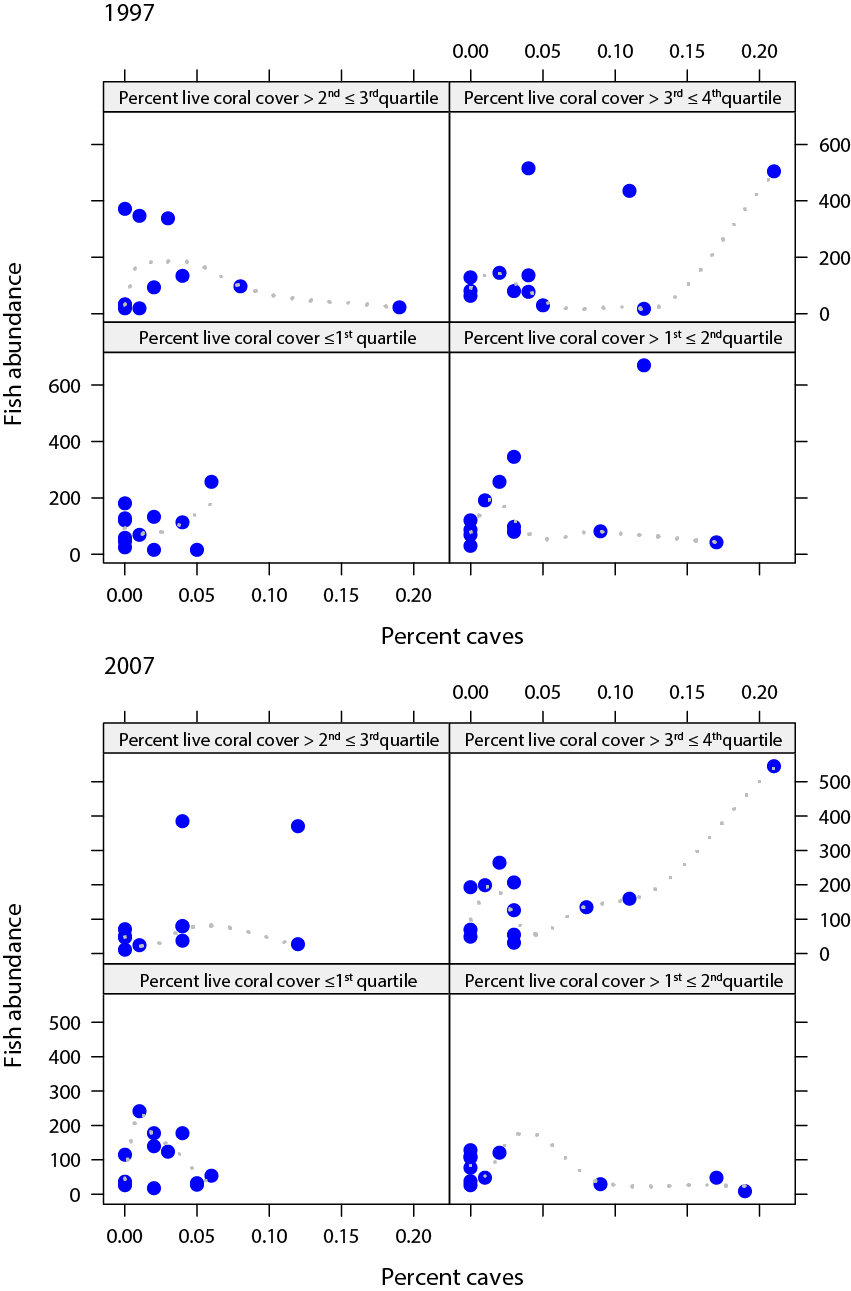
**

**Fig. S2** **A)** The relationship between patch volume and log fish abundance at difference levels of the continuous predictor ‘percent caves’ (indicated by the orange vertical bar at the top of each graph with values of percent cave increasing from left to right, bottom to top). Graphs display model predictions obtained with the R package effects (Fox et al. 2014). The grey area is the 95% confidence interval. Vertical black lines indicate the range of data points used in the model. **B)** The same relationship displayed for the raw, untransformed data, for each year sampled (1997 and 2007). N.B. Panels in this figure represent different levels of patch volume rather than percent caves.

**A)**


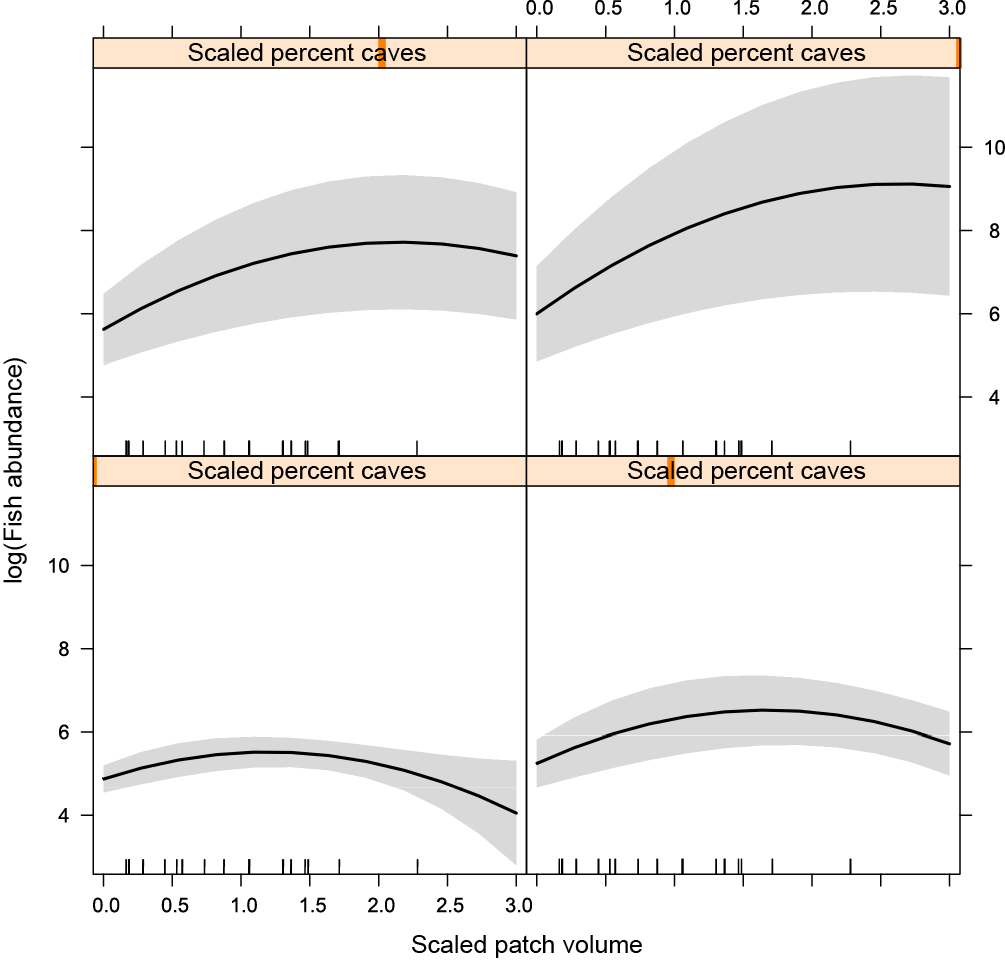


**B)**

**
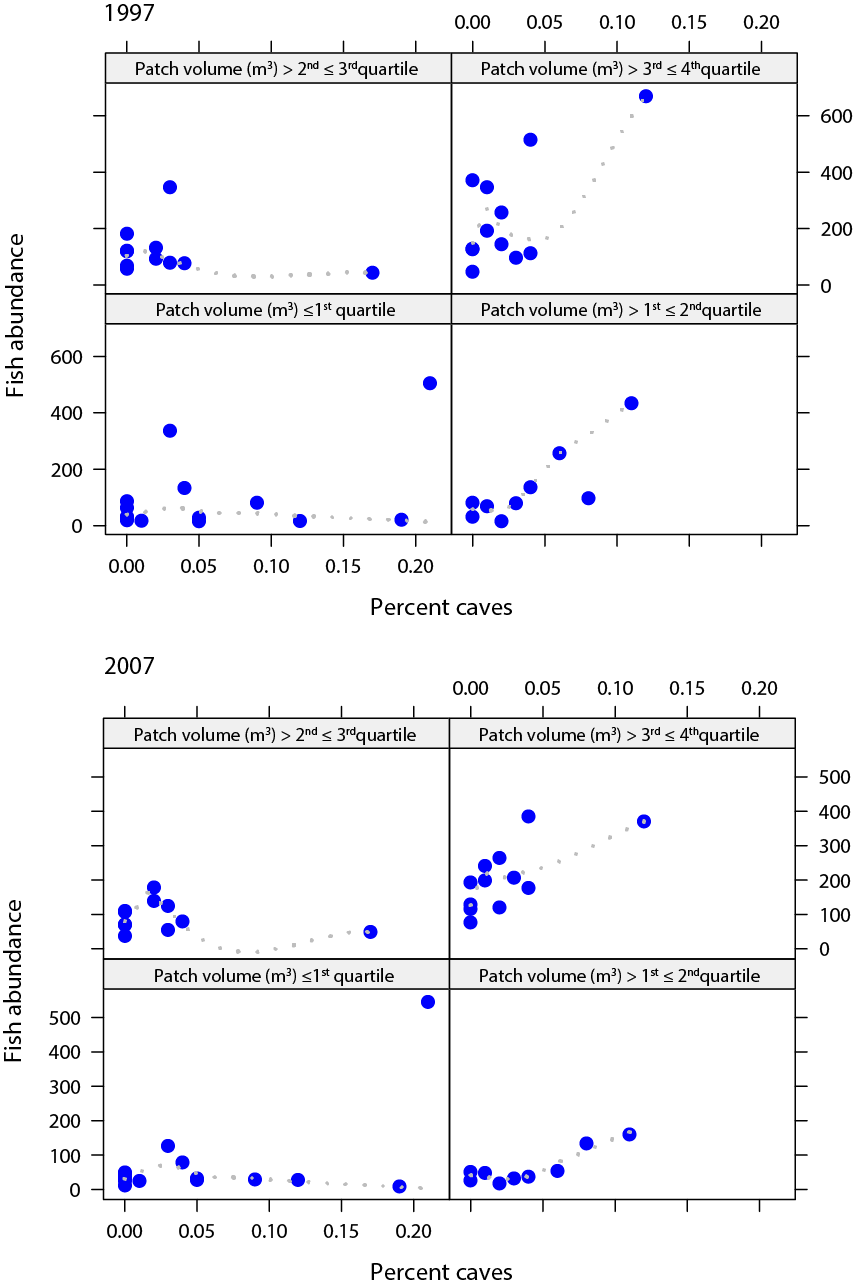
**

**Fig. S3** **A)** The relationship between fish species richness and abundance of the cleaner fish *Labroides dimidiatus*. The predictor *L. dimidiatus* abundance is scaled (i.e. a one-unit difference represents a difference of one-standard deviation). The graph displays model predictions obtained with the R package effects (Fox et al. 2014). The grey area is the 95% confidence interval. Vertical black lines indicate the range of data points used in the model.  **B)** The same relationship displayed for the raw data, for each year sampled (1997 and 2007).

**A)**

**
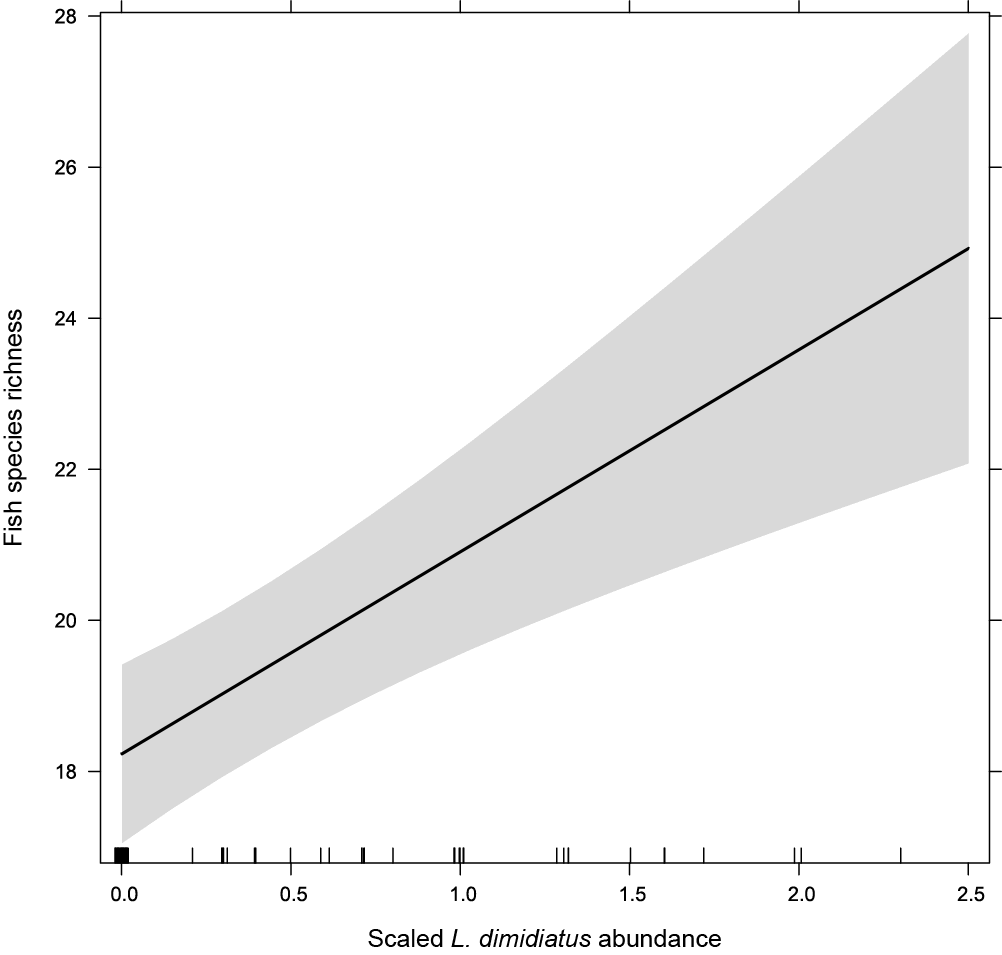
**

**B)**

**
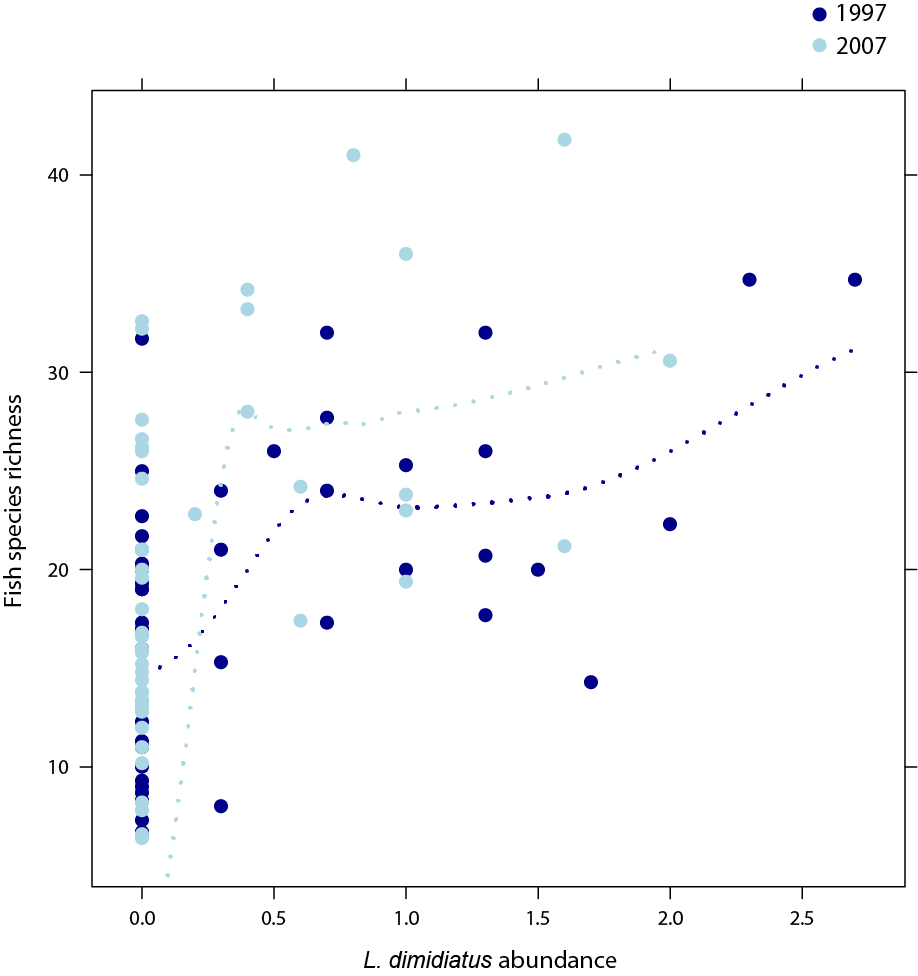
**

**Fig. S4** **A)** The relationship between patch volume and fish species richness at difference levels of the continuous predictor ‘percent caves’ (indicated by the orange vertical bar at the top of each graph with values of percent cave increasing from left to right, bottom to top). Graphs display model predictions obtained with the R package effects (Fox et al. 2014). Predictors are scaled (i.e. a one-unit difference represents a difference of one-standard deviation). The grey area is the 95% confidence interval. Vertical black lines indicate the range of data points used in the model. **B)** The same relationship displayed for the raw data, for each year sampled (1997 and 2007). N.B. Panels in this figure represent different levels of patch volume rather than percent caves.

**A)**


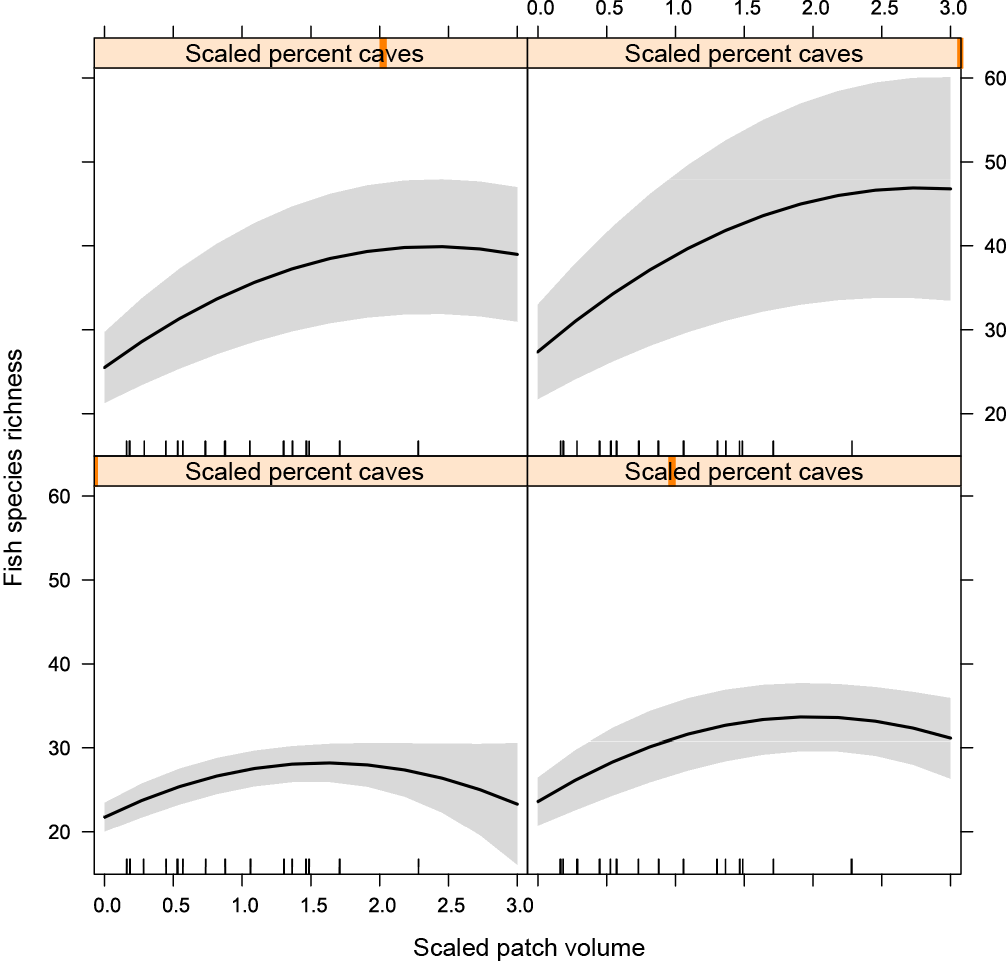


**B)**


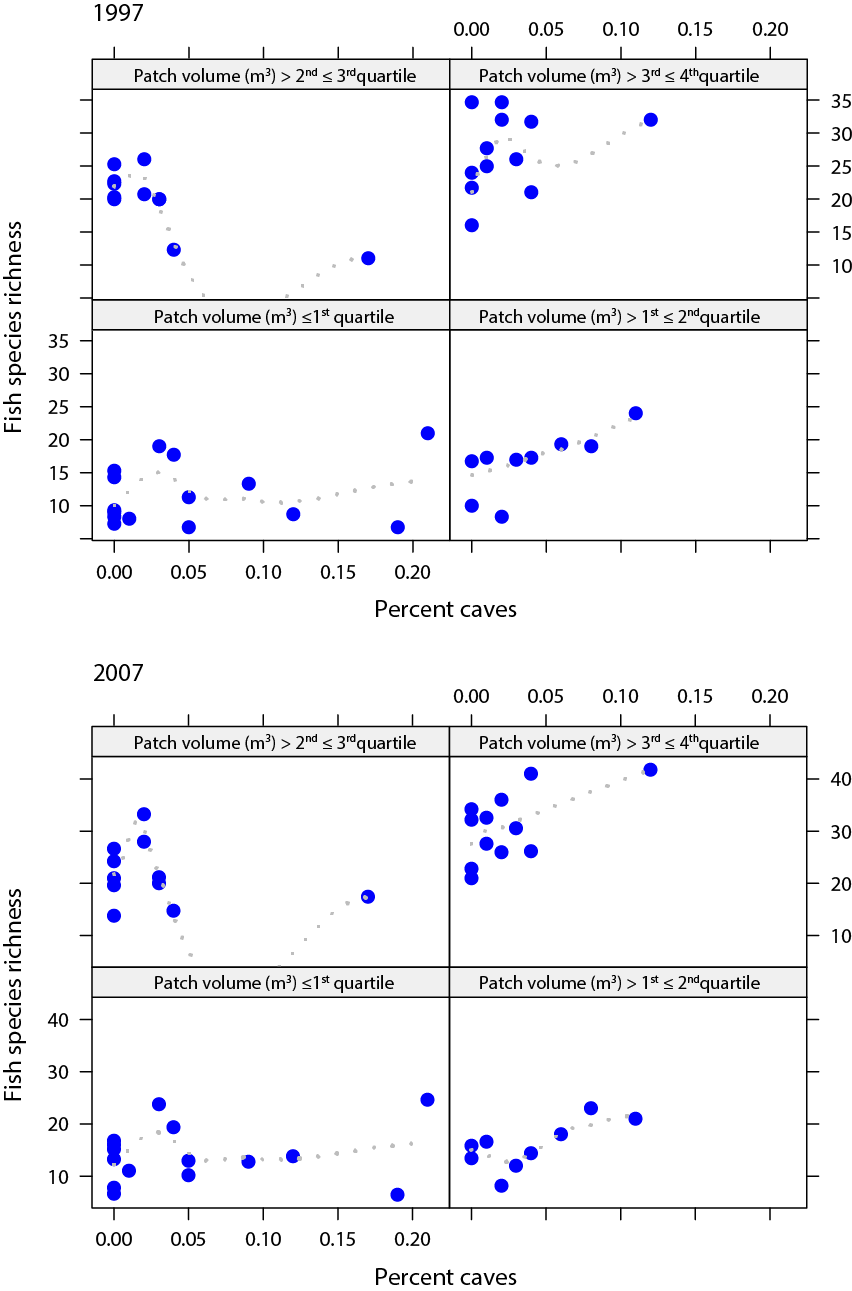


**Fig. S5** **A)** The relationship between patch volume and fish species richness at difference levels of the continuous predictor ‘percent holes’ (indicated by the orange vertical bar at the top of each graph with values of percent holes increasing from left to right, bottom to top). Graphs display model predictions obtained with the R package effects (Fox et al. 2014). Predictors are scaled (i.e. a one-unit difference represents a difference of one-standard deviation). The grey area is the 95% confidence interval. Vertical black lines indicate the range of data points used in the model. **B)** The same relationship displayed for the raw data, for each year sampled (1997 and 2007).

**A)**


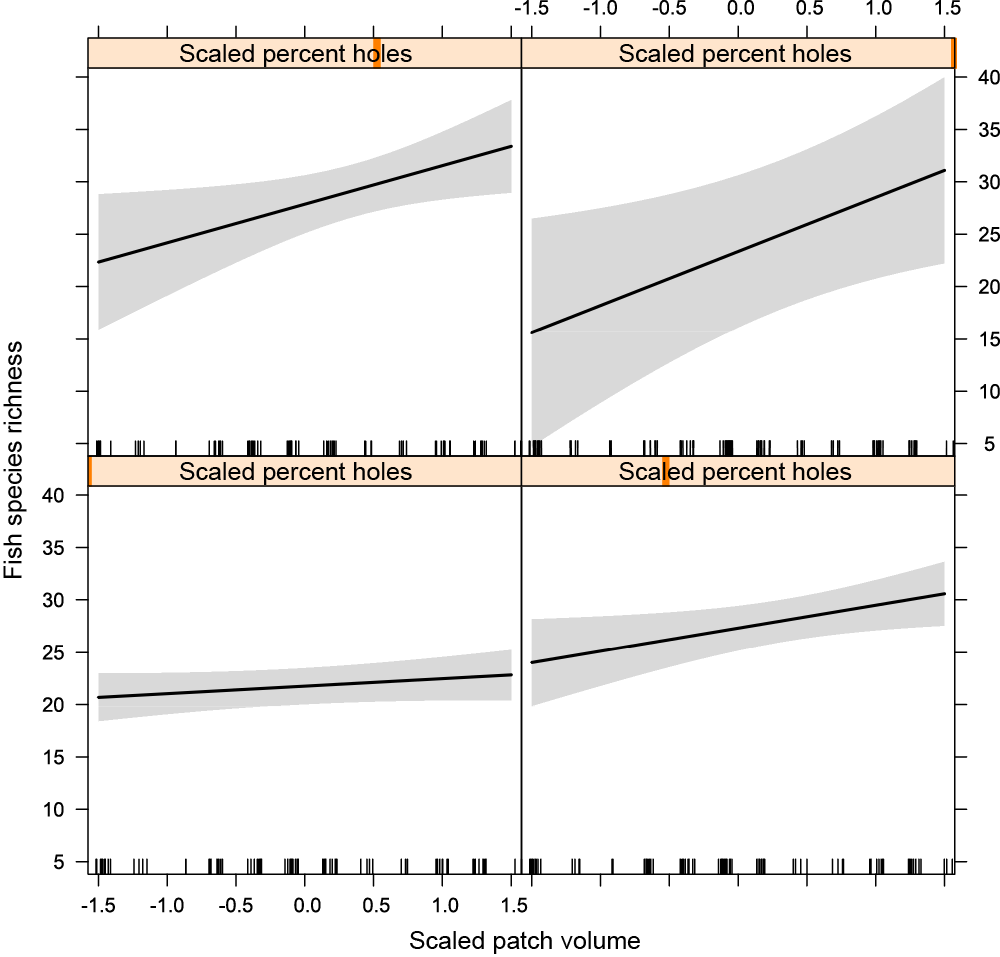


**B)**

**
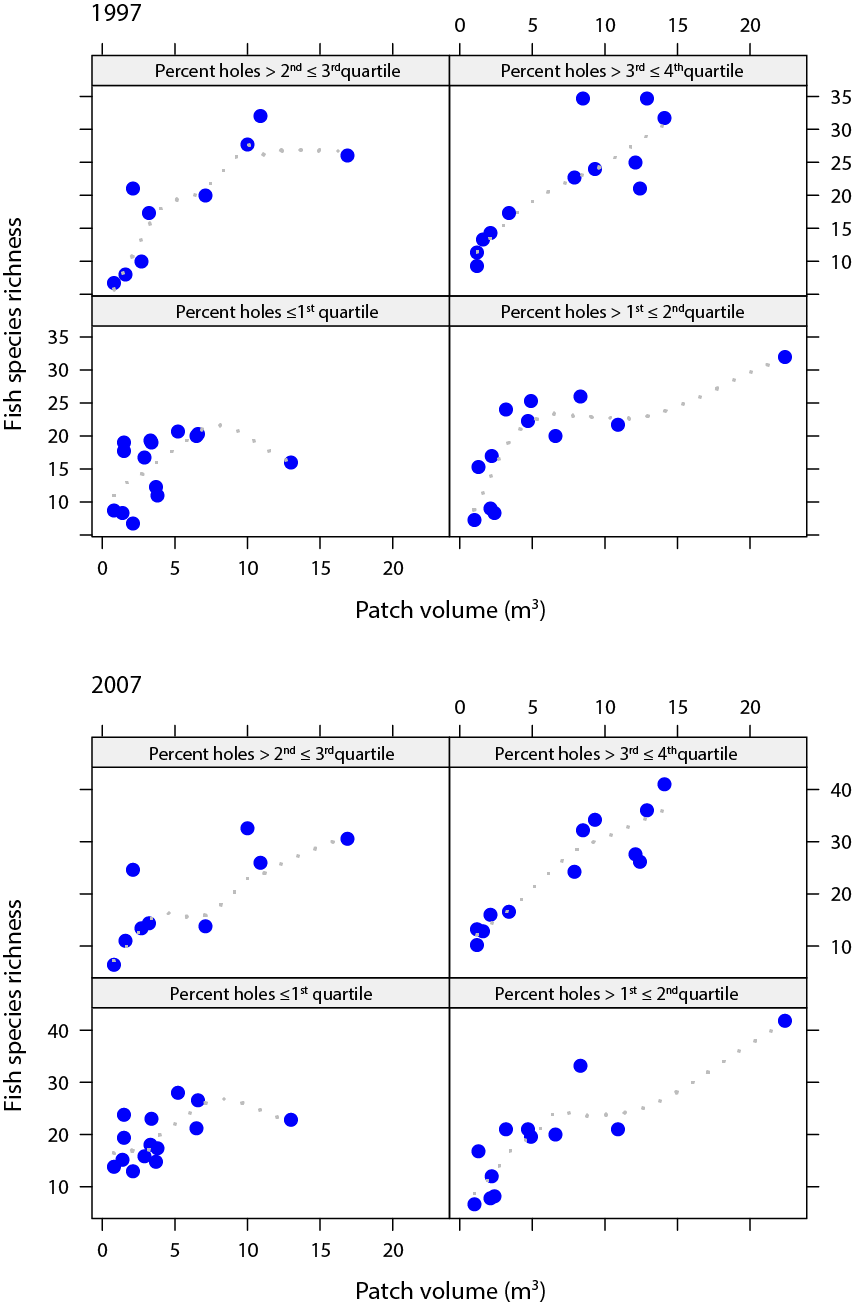
**

**Fig. S6** **A)** The relationship between percent live coral cover and fish species richness at difference levels of the continuous predictor ‘depth’ (indicated by the orange vertical bar at the top of each graph with values of depth increasing from left to right, bottom to top. Graphs display model predictions obtained with the R package effects (Fox et al. 2014). Predictors are scaled (i.e. a one-unit difference represents a difference of one-standard deviation). The grey area is the 95% confidence interval. Vertical black lines indicate the range of data points used in the model. **B)** The same relationship displayed for the raw data, for each year sampled (1997 and 2007).

**A)**


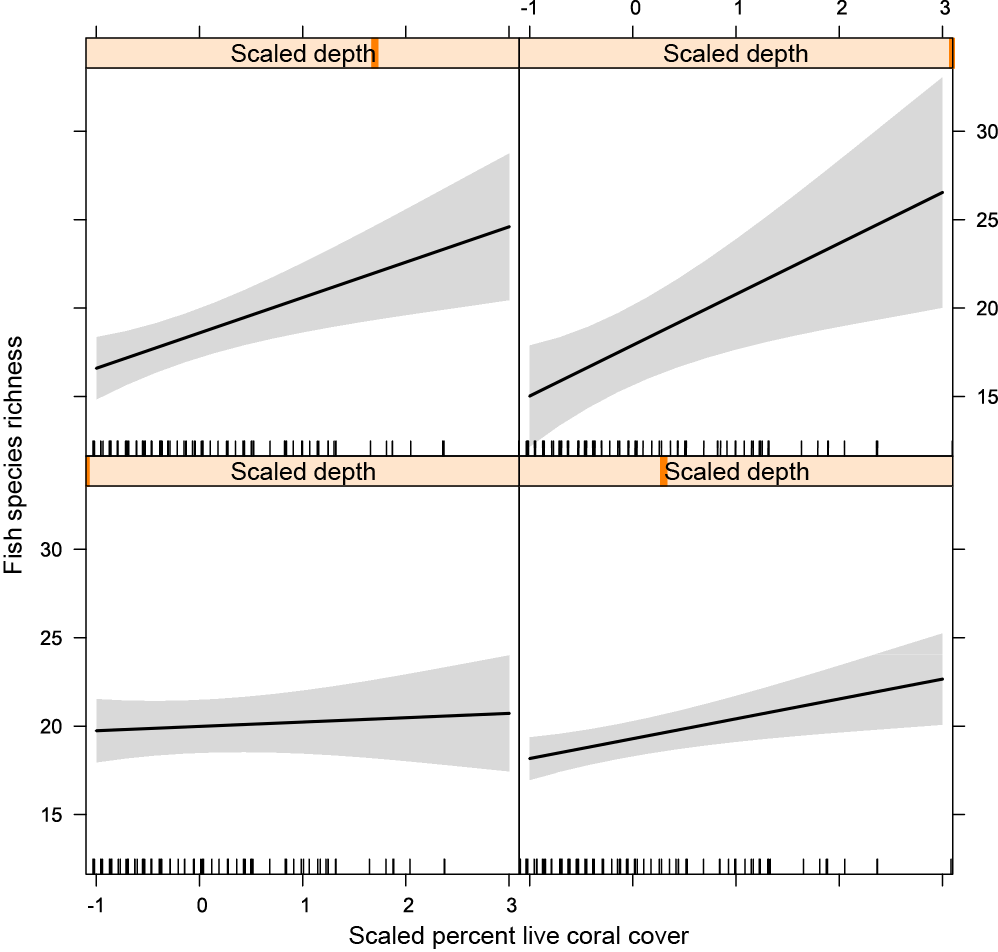


**B)**

**
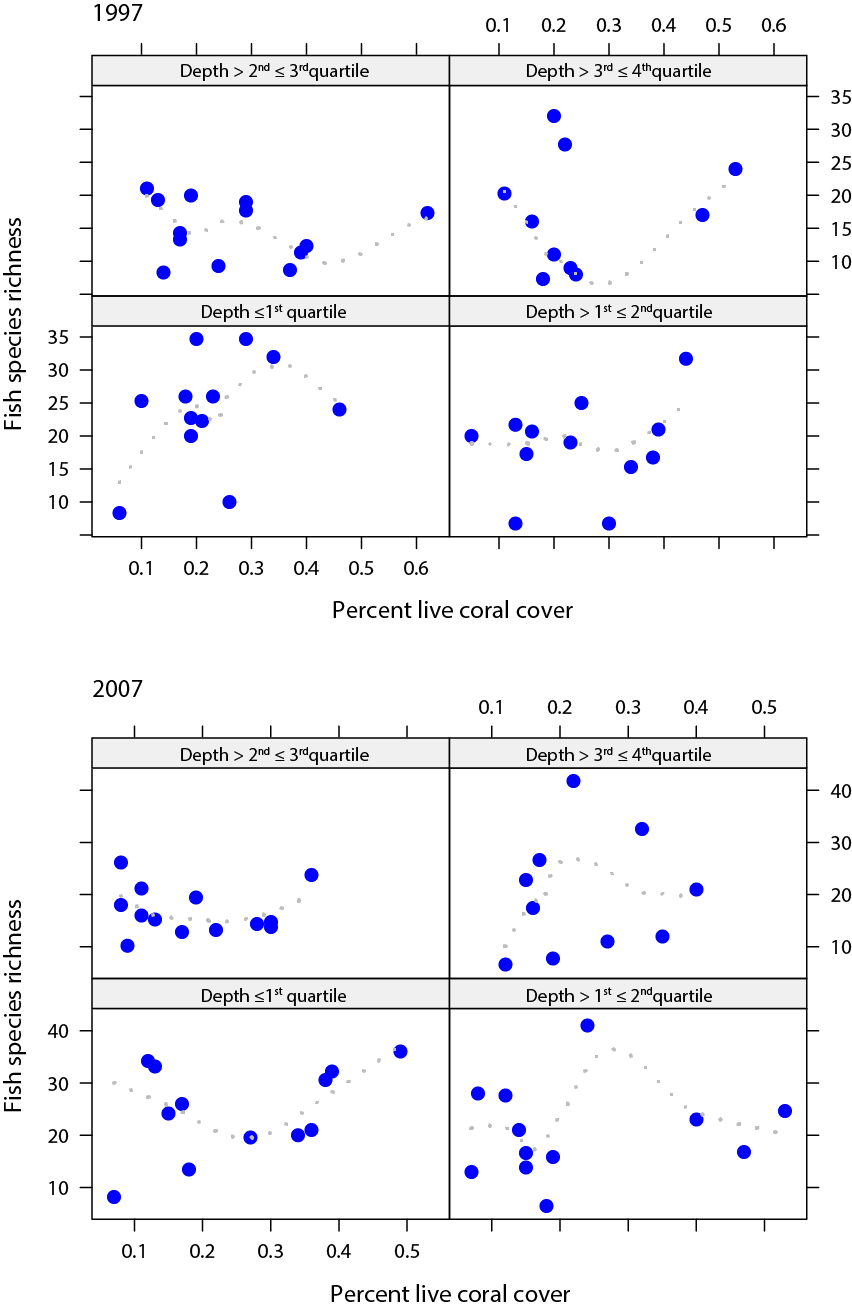
**
